# Calvarial Tuberculosis With Skin Tuberculosis in a Child: A Rare Case Report

**DOI:** 10.7759/cureus.52884

**Published:** 2024-01-24

**Authors:** Shikha Swaroop, Preeti Srivastava, Kumar Diwakar, Sumeet Biswal

**Affiliations:** 1 Pediatrics, Tata Main Hospital, Manipal Tata Medical College, Manipal Institute of Higher Education (MAHE), Jamshedpur, IND

**Keywords:** tuberculosis verrucosa cutis, skeletal tuberculosis, tuberculous meningitis, skin tuberculosis, calvarial tuberculosis

## Abstract

Calvarial tuberculosis (TB) is an uncommon form of skeletal TB. Early diagnosis can be challenging as they may exhibit diagnostic dilemmas. Another rare kind of skin TB is called TB verrucosa cutis. In this case, both of these uncommon forms were observed simultaneously and were effectively treated with first-line antitubercular therapy.

## Introduction

Infection with *Mycobacterium tuberculosis* (TB) is widespread in developing nations. In developed countries, tuberculosis (TB) cases have also increased due to the resurgence of immune compromised conditions, such as human immunodeficiency virus (HIV) infection. As per the global TB report of the World Health Organization (WHO) in 2022, among all TB cases, 6.7% were among people living with HIV. Geographically, most TB cases in 2021 were in the WHO regions of Southeast Asia (45%), Africa (23%), and the Western Pacific (18%), with smaller shares in the Eastern Mediterranean (8.1%), the Americas (2.9%), and Europe (2.2%). Both skin TB and calvarial TB are uncommon. Calvarial TB, a rare form of skeletal TB affecting the skull bones, has a reported prevalence of up to 3.57% of skeletal TB [1]. Roughly 1.5% of extrapulmonary TB cases are attributed to cutaneous TB [2].

Calvarial TB typically begins in the diploic space and progressively spreads to the inner and outer tables [3]. The condition manifests as subgaleal swelling with discharging sinuses if the outer table is affected. The deposition of extradural granulation tissue occurs if the inner table is compromised. It can affect the meninges and spread past the duramater, potentially resulting in meningitis if left undiagnosed. Timely radiological investigations and sound clinical judgement are essential for the diagnosis. The frontal and parietal bones show punched-out lesions on an X-ray and computed tomography (CT) scan.

Here, we report a rare case of calvarial TB that spread to cause tuberculous meningitis (TBM) with coexisting cutaneous TB.

## Case presentation

A 10-year-old boy presented with a seven-day history of vomiting and fever. Upon additional assessment, the parents reported that the fever started gradually, and his vomiting aggravated further over the past three days. Fever was low grade continuous with one spike of temperature in a day. Vomiting was projectile in nature, with one to two episodes in a day initially, which increased to four to five episodes at the time of admission. A year and a half ago, he experienced headaches, for which a CT scan of his head was done. Headaches at that time were dull aches in the frontal region, aggravated at morning. CT head report suggested frontal bone erosion with hypodense collection in the scalp sign of osteomyelitis. According to the patient's father, some injections were given for the child's condition, but the exact prescription was not available. The patient belonged to an eastern Indian tribal family with a low socioeconomic status. There was no family history of TB or TB contact. There was no history of staying in crowded places, such as refugee camps. The child's vital signs were normal upon general examination, and a Bacillus Calmette-Guerin (BCG) vaccine scar was visible. Swelling was seen in the right submandibular and supraorbital areas. Over the left knee, there was a rough single verrucous plaque over the skin. During a central nervous system (CNS) examination, the patient was conscious but spoke incoherently. The Glasgow coma scale was 14. Neck stiffness was present, tone was increased in all four extremities, and bilateral plantar was extended. Cranial nerve examination results were normal. There was no significant lymhadenopathy or hepatosplenomegaly. Dilated fundoscopy was normal. Pus aspiration was done from submandibular swelling, and fluid sent for culture was sterile. Cerebrospinal fluid (CSF) examination showed neutrophilia with high protein and low blood glucose (Table 1). CSF culture and blood culture were sterile.

**Table 1:** Serial cerebrospinal fluid examination CSF: cerebrospinal fluid, WBC: white blood cell, RBS: random blood sugar

Investigation	Day 1	Day 9	References
CSF appearance	Hazy	Clear	Clear
Total WBC per cumm in CSF	160	140	0-5
CSF lymphocyte %	30	100	100
CSF neutrophils %	70	0	0
Microorganism	Not found	Not found	None
Protein in CSF mg/dl	221	197.6	20-30
Sugar in CSF mg/dl	11	27	60-80

**Figure 1:**
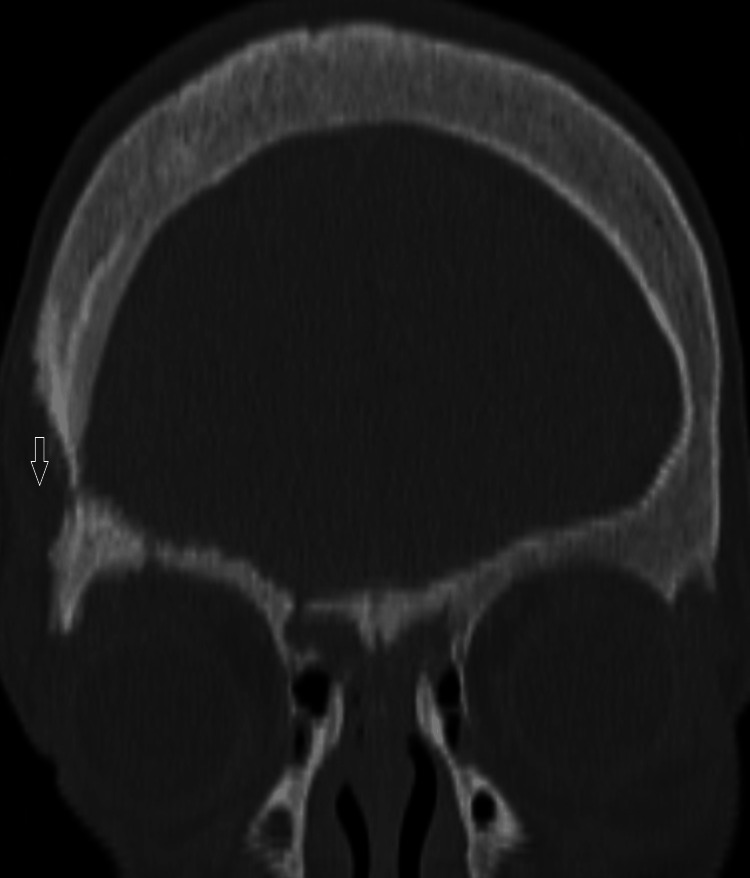
Right parietal bone erosion

The patient was started on empirical intravenous antibiotic (ceftriaxone and vancomycin), suspecting pyogenic meningitis. There was no improvement in the fever spike and sensorium of the patients after seven days of antibiotics. The patient remained hemodynamically stable. A repeat CSF examination was done, which showed 100% lymphocytes with elevated protein and low glucose (Table 1). A repeat CSF culture sent was sterile. The lesion on the left knee appeared to be tuberculous verrucous cutis, as per the dermatologist's assessment. A contrast-enhanced CT scan of the brain revealed osteolytic lesions of multiple skull bones with some granulations and a small extracalvarial collection in the right frontal bone with a normal brain parenchyma, most likely of infectious origin (Figure 1). Additional testing was performed for TB, including a chest X-ray and nebulized sputum for the cartridge-based nucleic acid amplification test (CBNAAT). While the X-ray of the chest was normal, the nebulized sputum tested positive for CBNAAT. The patient was started on antituberculosis treatment (ATT) based on repeat CSF findings, skin lesion, and CBNAAT positive sputum. The patient's fever gradually subsided, and ATT was planned to be administered for at least a year. Two months of four-drug (isoniazid, rifampicin, ethambutol, and pyrazinamide) intensive phase and 10 months of three-drug (isoniazid, rifampicin, and ethambutol) continuation phase was administered.

**Figure 2:**
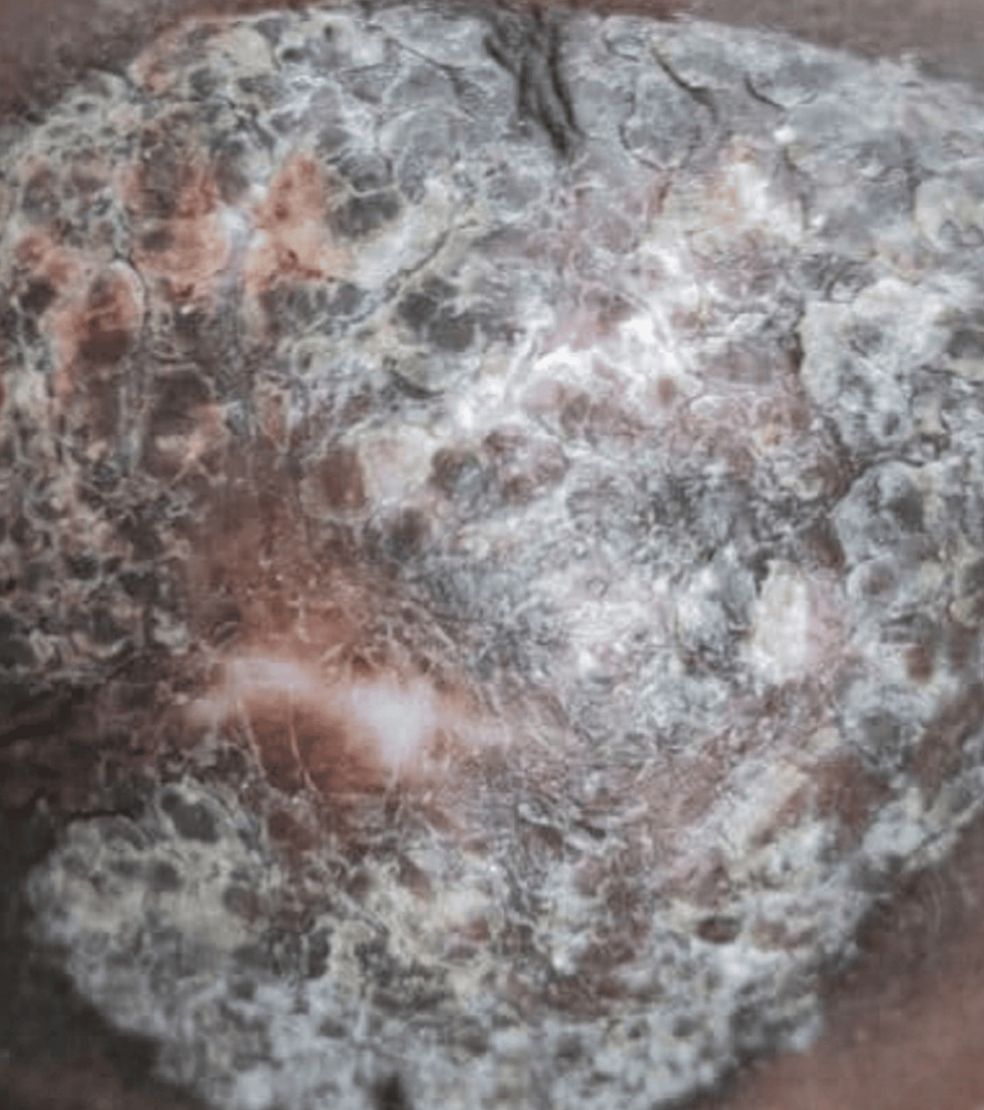
Skin tuberculosis (tuberculosis verrucosa cutis)

**Figure 3:**
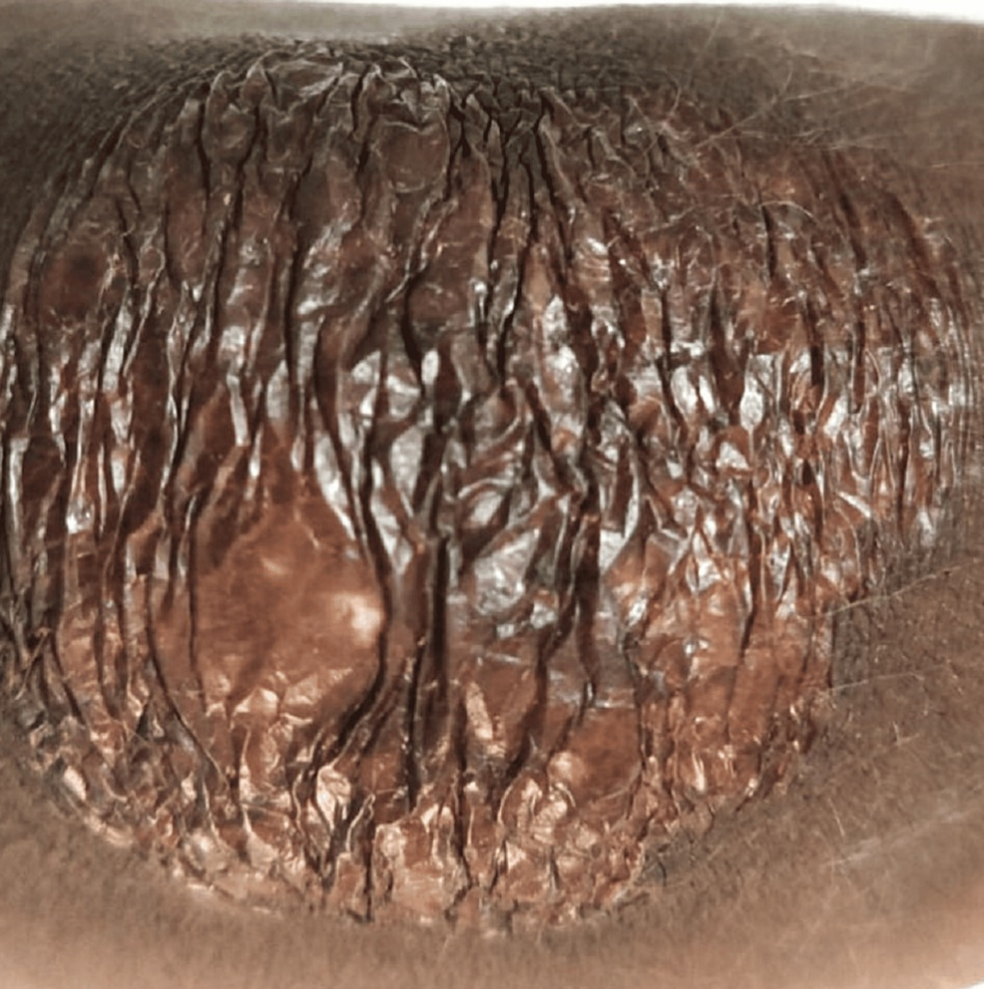
Healing skin tuberculous lesion in the patient post 45 days of the antituberculosis treatment

After a month and a half of starting ATT, the patient was readmitted for a single episode of generalized tonic clonic convulsion, which was managed with phenytoin parenteral therapy followed by oral and discharged once stable. Seizure was presumed to be sequelae of TBM. It was planned to give antiepileptic (phenytoin) for at least two years. The patient's overall condition improved upon follow-up, and the skin lesion demonstrated significant improvement (Figures 2, 3). The patient has been under routine follow-up care. He is asymptomatic after completing the ATT course.

## Discussion

Most cases of calvarial TB occur in people under the age of 20 [3]. Most affected are the parietal and frontal bones [3]. Such a disease is seen in immunocompetent patients. There is a direct hematogenous spread of bacteria from the primary focus, as lymphatic vessels in the skull bones are sparse [4]. The TB bacteria grow slowly in the diploic space of the bone, causing osteolytic lesions in the bone structure. Both the inner and outer tables may become infected. The outer table is affected commonly [5]. The dura mater prevents brain dissemination. The inner table is eventually affected. Calvarial TB takes a long time to spread across the dura mater, affecting the meninges, and it is also extremely rare [6].

In our case, the patient experienced headache symptoms about a year and a half ago, for which he was investigated and treated. However, at that time, the diagnosis of TB was missed. Gradually, the infection spread extradurally, causing orbital swelling, and intradurally, causing meningitis. The fact that the sputum for CBNAAT was positive with normal X-ray chest findings was intriguing. Tuberculous verrucous cutis is a rare form of cutaneous TB caused by *Mycobacterium tuberculosis* being directly inoculated into the skin after a minor trauma. In our case, the patient was a member of a native tribal population who, because of their way of life, suffered from mild traumas regularly. In this case, it is difficult to state whether skin TB was a result of the spread of bacteria from the focus at calvarium or was a result of a direct inoculum. This case was a rare TB amalgam, with both calvarial TB leading to TBM and TB verrucous cutis occurring concurrently. The patient responded well to medical management, as there was no large intracranial collection requiring surgical intervention.

The diagnosis of skull TB based on neuroimaging is difficult because it mimics other diseases, such as malignancy and osteomyelitis. In cases where there is no other focus of the disease and CSF is normal, a skull biopsy of the lesion is required to confirm the diagnosis, which necessitates good infrastructure. Initially, the patient's symptoms are mild, such as a headache. As a result, early detection of skull TB is difficult.

The presence of neutrophilia in the CSF has been reported in early cases of tuberculous meningitis [7]. This may cause clinicians to be confused about pyogenic meningitis [7]. In our case, the patient had calvarial TB for a long time, as evidenced by previous hospital admission for headache, but he presented to us with a seven-day acute febrile illness. This is probably when the TB bacteria infiltrated the dura and began to affect the meninges. After seven days, a CSF examination revealed lymphocytic pleocytosis with the sugar and protein characteristic of TBM.

TB verrucous cutis is a paucibacillary form that develops in immunocompetent individuals after exogenous skin inoculation with TB bacteria. Such skin TB is uncommon to see. This is a clinical diagnosis from an experienced clinician [8]. A skin biopsy may be done in case of doubt. In this case, the lesion typically appeared as TB verrucous cutis and so was diagnosed clinically.

## Conclusions

Calvarial TB and skin TB, such as TB verrucous cutis, are uncommon extrapulmonary forms of TB even in endemic countries, such as India. A TBM CSF picture in the early stages may resemble pyogenic meningitis. A good clinical acumen and awareness are required for an early-stage diagnosis and timely treatment.
